# Allergen immunotherapy

**DOI:** 10.1186/1824-7288-40-S1-A79

**Published:** 2014-08-11

**Authors:** Giuseppe Crisafulli, Lucia Caminiti, Fernanda Chiera, Vincenzo Ramistella, Stefania Arasi, Giovanni Battista Pajno

**Affiliations:** 1 Department of Pediatrics, Allergy Unit- University of Messina- Italy

## 

Allergen Immunotherapy (AIT), is effective in reducing the clinical symptoms associated with allergic rhinitis, asthma and venom induced anaphylaxis [1]. Subcutaneous (SCIT) and Sublingual (SLIT) with unmodified allergen extracts are the two routes of administration of allergen vaccines.

**Figure 1:**
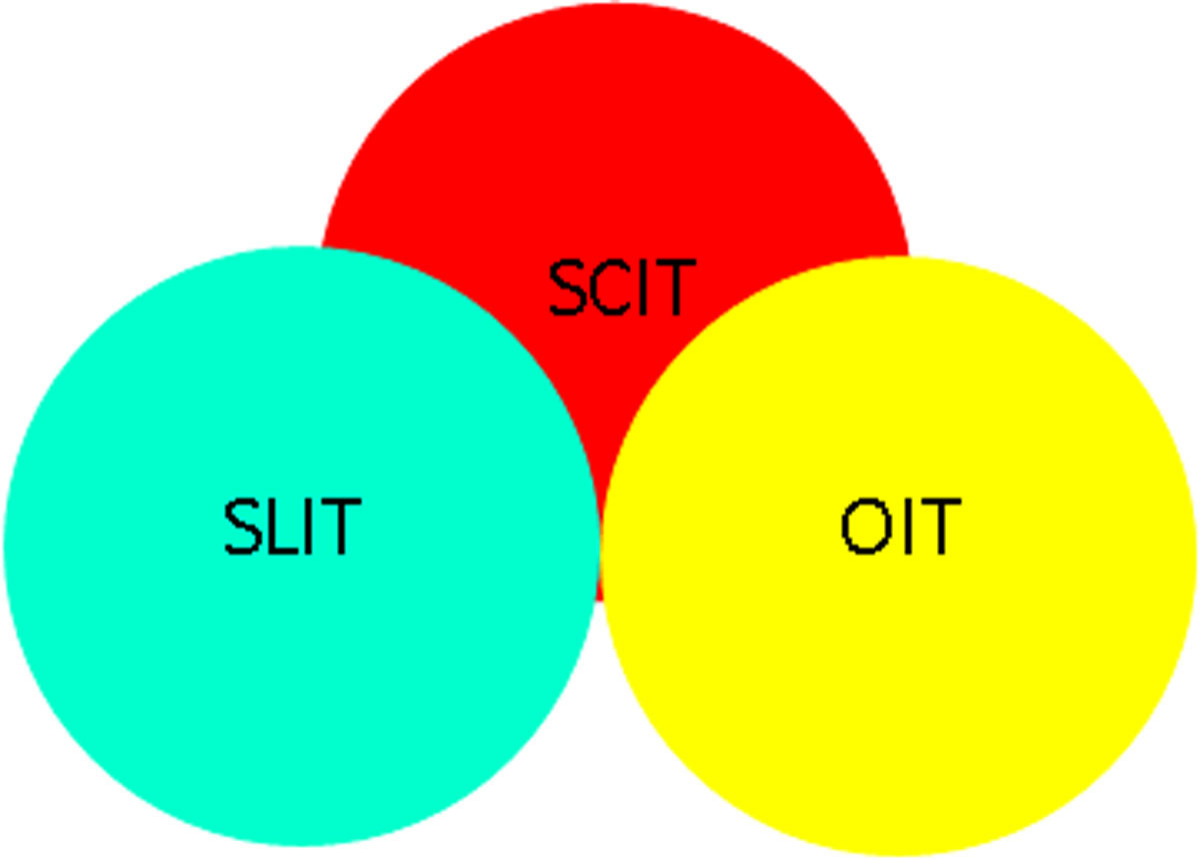
**The immunotherapy’s triad.** The main routes and forms of immunotherapy. Due to the complexity of IgE mediated disorders the triad could be considered as complementary therapy. Thus SCIT, SLIT and OIT could be used in various combination and timing in order to optimize both adherence to treatment and therapeutics effect. SCIT= subcutaneous immunotherapy for the treatment of allergic asthma and allergic rhinitis. SLIT= sublingual immunotherapy for the treatment respiratory allergies and IgE mediated food allergies. OIT= oral immunotherapy for the active treatment of IgE mediated food allergy. *On the horizon: the treatment of extrinsic form IgE mediated atopic dermatitis.*

In addition, AIT has been positioned as the only treatment that may alter the natural course of allergic disease [2]. However both SCIT and SLIT require that the treatment is taken regularly over several years e.g. monthly in a supervised medical setting with SCIT and at lowest three times a week with SLIT. Emerging evidence suggests that specific allergen immunotherapy may be effective in other allergic conditions such as IgE mediated food allergy [3,4] and extrinsic form IgE mediated Atopic Dermatitis [5]. On all these fields, the immunotherapy’s triad can be an effective tool (Figure 1).

Moreover due to the complexity of IgE mediated disorders, each component of triad: SCIT, SLIT or Oral Immunotherapy (OIT) could be considered as complementary or synergic therapy. Currently, in paediatrics, the challenge is represented by the possibility of defeat the reluctance to encourage the implementation of early intervention in IgE mediated allergic diseases, with the goal of achieving either secondary prevention or long lasting benefit through immunotherapy(ies) which is the only antigen specific immunomodulatory treatment routinely available .These effects are of particular relevance in paediatric population with the aim of impairing the natural history of allergic diseases.

The mechanisms of action of AIT have been elucidated: a diminished allergen specific T-cell proliferation and suppressed secretion of TH2 cell responses are the characteristic hallmarks. In addiction, T regulatory (Treg) cells inhibit the development of allergen specific TH2 and TH1 cell responses an therefore exert key roles in healthy immune response to allergens. Treg cells potently suppress IgE production and directly or indirectly control the activity of effectors cells of allergic inflammation, such as eosinophils, basophils, and mast-cells [6].

Therefore, AIT in different forms represent an effective therapeutic approach in children with IgE mediated respiratory disorders. Moreover, in other allergic disorders that are no part of the respiratory disease spectrum, the evidence is beginning to emerge that these diseases also will respond to allergen specific immunotherapy.
